# TRIM32 reduced the recruitment of innate immune cells and the killing capacity of *Listeria monocytogenes* by inhibiting secretion of chemokines

**DOI:** 10.1186/s13099-023-00558-9

**Published:** 2023-07-06

**Authors:** Xuan OuYang, Peng Liu, Yuling Zheng, Hua Jiang, Qingyu Lv, Wenhua Huang, Huaijie Hao, Yaya Pian, Decong Kong, Yongqiang Jiang

**Affiliations:** 1 State Key Laboratory of Pathogen and Biosecurity, Institute of Microbiology and Epidemiology, Beijing, China; 2 National Center for Clinical Laboratories, Institute of Geriatric Medicine, Chinese Academy of Medical Sciences, Beijing Hospital/National Center of Gerontology, Beijing, China

**Keywords:** TRIM32, *Listeria monocytogenes*, Sepsis, Innate immune, iNOS

## Abstract

*Listeria monocytogenes* (*Lm*) is a facultative, intracellular Gram-positive pathogenic bacterium that causes sepsis, a condition characterized by persistent excessive inflammation and organ dysfunction. However, the pathogenesis of *Lm-*induced sepsis is unknown. In this research, we discovered that TRIM32 is required for innate immune regulation during *Lm* infection. *Trim32* deficiency remarkably reduced bacteremia and proinflammatory cytokine secretion in mice with severe *Lm* infection, preventing sepsis. *Trim32*^−/−^ mice had a lower bacterial burden after *Lm* infection and survived significantly longer than wild-type (WT) mice, as well as lower serum levels of inflammatory cytokines TNF-α, IL-6, IL-18, IL-12p70, IFN-β, and IFN-γ at 1 day post infection (dpi) compared to WT mice. On the other hand, the chemokines CXCL1, CCL2, CCL7, and CCL5 were enhanced at 3 dpi in *Trim32*^−/−^ mice than WT mice, reflecting increased recruitment of neutrophils and macrophages. Furthermore, *Trim32*^−/−^ mice had higher levels of macrophage-associated iNOS to kill *Lm*. Collectively, our findings suggest that TRIM32 reduces innate immune cells recruitment and *Lm* killing capabilities via iNOS production.

**Graphical Abstract:**

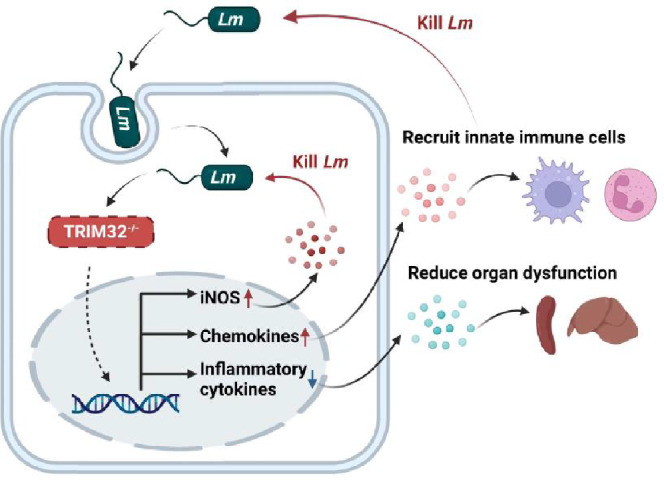

**Supplementary Information:**

The online version contains supplementary material available at 10.1186/s13099-023-00558-9.

## Introduction

*Listeria monocytogenes* (*Lm*) is a Gram-positive pathogenic bacterium that can cause a variety of clinical syndromes such as sepsis, meningitis, and rhombencephalitis [1]. The infection of mice with this pathogen in the laboratory provides a wealth of information about the roles of immune cells and cytokines in host immunity against this pathogen. Over the last 20 years, the use of *Lm* as an intracellular pathogen model to investigate the host immune responses has established a paradigm in infection biology and basic microbiology [2, 3].

The activation of the host’s innate immune system and inflammatory responses are required for effective *Lm* infection elimination [4]. *Lm* infection causes the release of pro-inflammatory cytokines [5], IFN-β and IFN-γ [6]. Mice investigations have demonstrated that in the absence of IFN-β signaling, animals cannot be effectively infected by *Lm*, and mice with greater IFN-β levels have a higher bacterial load [7, 8]. One of the key mechanisms for controlling and eliminating *Lm* infection at the early stages is the recruitment of myeloid cells such as neutrophils, macrophages, and dendritic cells (DCs) to the infected sites [9, 10]. Macrophages produce inducible nitric oxide synthase (iNOS), which is primarily regulated by the NF-κB and JAK-STAT1 signaling pathways, with the goal of eradicating *Lm* proliferation [11–13].

TRIM32 is a member of the tripartite motif family, which is characterized by the presence of a RING domain that confers E3 ubiquitin ligase activity and functions as an important immune-regulatory protein in response to pathogen infection [14]. TRIM32 has been shown to promote IFN-β secretion during herpes simplex virus infection via the STING-IRF3 signaling pathway in order to eliminate infection [15]. Furthermore, it also increased IFN-γ production in response to *Streptococcus suis* infection, influencing blood–brain barrier permeability and aiding bacterial clearance in the brain [16]. However, the roles of TRIM32 in immunoregulation in Gram-positive intracellular bacterial infections such as *Lm*, on the other hand, are unknown.

In this study, we discovered that TRIM32 deficiency may promote the synthesis of iNOS and increase the secretion of chemokines helpful for neutrophil and macrophage recruitment while decreasing the production of inflammatory cytokines to prevent organ dysfunction to kill *Lm*. The information presented here will provide further insights into the regulation of antimicrobial innate immunity.

## Materials and methods

### Bacterial strains, growth medium, and cell culture

The strain *Lm* 10403S used in this study was originally isolated from skin lesions in Bozeman, Montana, USA, and has previously been used in intracellular pathology experimental models [7, 17]. Single colonies were inoculated into 5 ml of BHI (brain–heart infusion) medium and shaking vigorously overnight at 37 °C. Working cultures were created by inoculating 1% dilutions of overnight cultures into new BHI medium and culturing for 3 h at 37 °C to mid-log phase (about 5 × 10^8^ CFU/ml).

Thioglycolate-elicited peritoneal macrophages (PMs) from C57BL/6 mice were prepared and cultured in endotoxin-free RPMI-1640 medium with 10% (vol/vol) FBS (Invitrogen). Bone marrow-derived macrophages (BMDMs) from C57BL/6 mice were cultured in endotoxin-free DMEM (Gibico) supplemented with 10% FBS after being prepared and generated in M-CSF (30 ng/ml) for 7 days (Peprotech). THP-1 cells were cultured in endotoxin-free RPMI-1640 medium with 10% (vol/vol) FBS (Invitrogen).

### Ethics statement

All animals used in this study were housed at the Academy of Military Medical Sciences (AMMS) animal center in Beijing, China. Animals were cared for in accordance with the approved principles of laboratory animal care in China. All experimental procedures were approved by the Institutional Animal Care and Use Committee of the AMMS (IACUC-DWZX-2021-023).

### Trim32-deficient mice

*Trim32*-deficient C57BL/6 mice were generated as previously described [16] using transcription activator-like effector nuclease (TALEN). Briefly, exon 2 of the *trim32* gene (Ensembl accession no. ENSMUSG00000051675) was chosen as a TALEN target site to create *trim32*-deficient C57BL/6 mice. TALEN mRNAs generated by in vitro transcription were injected into fertilized eggs to produce knockout mice. The founders were genotyped using PCR and DNA sequencing, and the positive testers were bred to the next generation.

### Lm infection

The bacteria were collected at the mid-logarithmic growth stage, washed and re-suspended in PBS, and counted on agar plates. To evaluate mice survival rates, 1.0 × 10^5^ CFUs were injected intraperitoneally (i.p.). Meanwhile, mice were injected i.p. with 1.0 × 10^6^ CFUs to assess bacterial burden in the spleen and liver by plate counting, organ lesions by hematoxylin and eosin (H&E) staining, and immune cell components by flow cytometry.

BMDMs and PMs were seeded in 24-well plates with 12 mm-coverslips over each well, followed by *Lm* infection at a multiplicity of infection (MOI) of 0.25. After discarding the *Lm* media, the mixture was incubated for 1 h with DMEM containing 50 μg/ml gentamicin. Bacterial counts were determined at 6 h post infection (hpi).

### Flow cytometry

Spleens were minced through a 70 μm sieve and leukocytes were obtained after lysing red blood cells with ACK buffer. Livers were minced through a 100 μm cell strainer and digested for 30 min at 37 °C with rotation in HBSS (Ca^2+^, Mg^2+^) buffer containing 1 mg/ml (335 U/mg) collagenase IV (Sangon Biotech, Shanghai, China) and 200 μg/ml (100 Kunitz U/ml) DNase I (Sangon Biotech, Shanghai, China). Liver leukocytes were separated by percoll gradient centrifugation (GE Healthcare, Freiburg, Germany). The cells were then resuspended in RPMI-1640 at 1 × 10^6^/ml, and surface staining was performed as previously described [16].

F4/80 and CD11b were used to stain macrophages, CD11c and CD11b for DCs, and Ly6G and CD11b for granulocytes. CD11c-FITC (N418; Biolegend), Gr1-PE (RB6-8C5; Biolegend), CD11b-PerCP-Cy5.5 (M1/70; eBioscience), F4/80-APC (BM8; Biolegend), and CD3-Pacific Blue (145-2C11; eBioscience) antibodies were utilized. As for intracellular iNOS-PE (W16030C; Biolegend) staining, the fixation and permeabilization steps were followed by Intracellular Fixation & Permeabilization Kit (eBioscience). Flow cytometric acquisition was performed with a BD FACSVerse flow cytometer, and data were analyzed using FlowJo software (TreeStar, Ashland, OR, USA).

### Western blot

1–2 × 10^6^ cells were resuspended in 150 μl RIPA lysis solution (Beyotime, Shang Hai, China) containing 1 tablet/8 ml EDTA-free complete protease inhibitor (Roche), 1 tablet/8 ml phosphatase inhibitor (Roche) and 1 mM PMSF (Sigma, Taufkirchen, Germany). After protein quantification, 5 × SDS loading buffer was added for 10 min at 95 to 100 ℃. Equal amounts of protein samples were separated by SDS-PAGE and transferred to nitrocellulose filter membrane. Blots were blocked with 5% BSA before being treated with primary antibodies, which were then followed with IRDye 800CW/680RD secondary antibodies (LI-COR Biosciences, USA). GAPDH (Sigma, Taufkirchen, Germany) and anti-TRIM32 (Abcam, Cambridge, UK) were employed. Western blot images were captured by the Odyssey SA infrared imaging system (LI-COR Biosciences, U.S.A.) and analyzed with Image Studio 5.x software (LI-COR Biosciences, America).

### ELISA

Inflammatory cytokines were measured using the Cytokine & Chemokine 20-Plex Mouse ProcartaPlex^™^ Panel 1 (Invitrogen) kit and the Luminex 200 system (Luminex Corporation, Austin, TX, USA). Furthermore, the levels of IFN-γ/IFN-β in the supernatants of serum, spleen, and liver were determined using ELISA kit (R & D Systems). The procedures were carried out in accordance with the kit’s protocol.

### Histopathology

Mice tissues were dissected and placed into 10% formalin-fixed liquid for H&E analysis. Sample sections were stained with H&E reagents according to the routine procedures. In brief, tissue samples were dehydrated in a graded series of alcohol washes, embedded in paraffin, sectioned into 4 μm slices, followed by H&E stain for a microscopic examination. Pictures were acquired using an Olympus BX53 microscope.

### Statistical analysis

Unless otherwise specified, all of the data in this research are expressed as the mean ± standard error of the mean (SEM). The data analysis was based on the data’s normality and homogeneity of variance. Log-rank (Mantel-Cox) test was conducted to determine statistical significance for survival curve data, while others were analyzed using an unpaired two-tailed Student’s t-test on the results from two groups or two-way ANOVA followed by Dunnett’s multiple comparisons teston the results from three or more groups. For all tests, a P value of < 0.05 was considered the threshold for significance. All statistical analysis in this study were performed with the GraphPad Prism 8.0.1 software program (GraphPad Software Inc, San Diego, CA, USA).

## Results

### Trim32 deficiency improved resistance against Lm infection

**Fig. 1:**
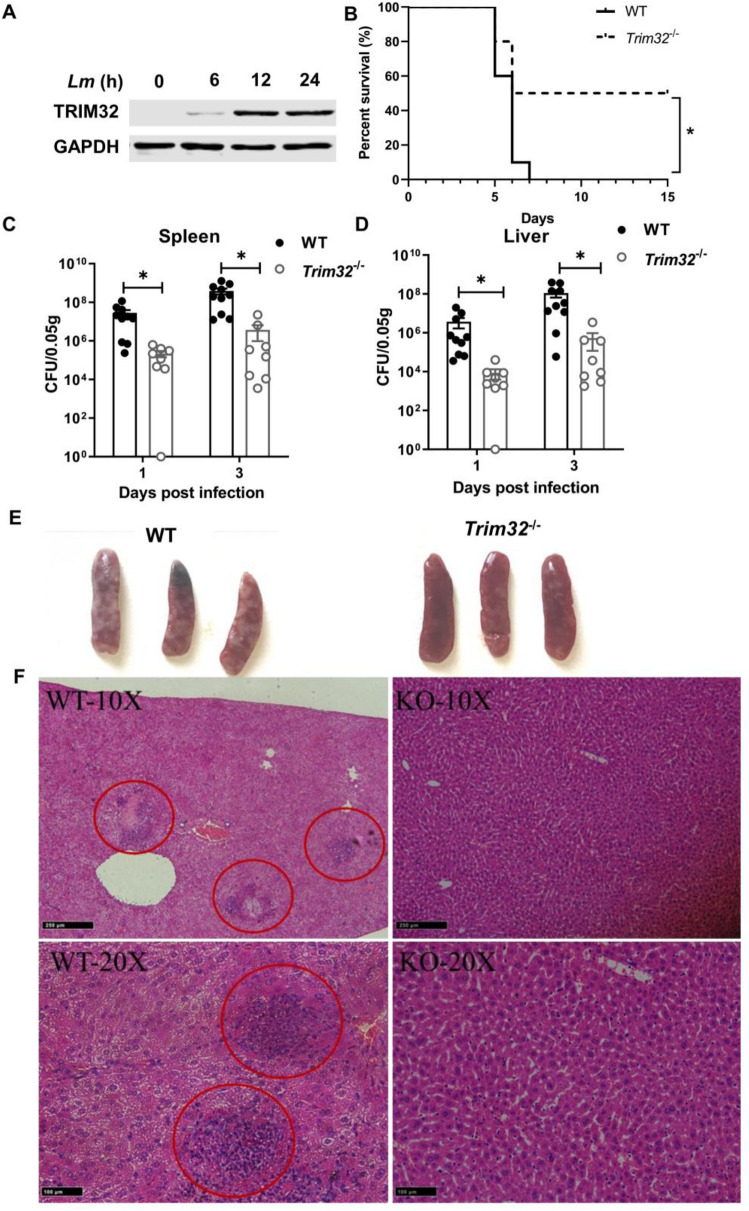
*Trim32* deficiency improved resistance against *Lm* infection. **A** Immunoblot analysis of inducible TRIM32 expression in THP-1 cells upon *Lm* infection. THP-1 was differentiated with 100 nM PMA for 2 days and replaced by RPMI media, followed by *Lm* infection at a MOI of 0.1 for indicated times. **B** Survival curves for WT and *Trim32*^−/−^ mice infected with *Lm*. Mice (n = 10 per group) were injected i.p. with 1.0 × 10^5^ CFU of *Lm*, and their survival was monitored daily. **C**, **D** Bacteria counts in the spleen and liver of mice infected i.p. with 1.0 × 10^6^ CFUs of *Lm*. Bacterial counts were determined by colony plate count. Showed were the combined results of two independent experiments with a total of 8 to 10 mice per experimental group and time point. Mean value for each experimental group was shown by a bar and each symbol represents one mouse. Macroscopic examination of the spleen **E** and HE staining of liver **F** from infected *Trim32*^−/−^ and WT mice, and the red circles indicated areas of erosion. Mice were injected i.p. with 1.0 × 10^6^ CFUs of *Lm*, spleens and livers were inspected at 3 dpi. Data were a representative of three independent experiments. Statistical significance was calculated by means of (B) log-rank Mantel-Cox test, (C, D) two-way ANOVA followed by Dunnett’s multiple comparisons test; * *P* < 0.05, ** *P* < 0.01

We used PMA-differentiated THP-1 cells to examine the host *trim32* gene’s response to *Lm* infection (Fig. 1A). TRIM32 steadily elevated from 0 to 12 hpi and maintained it at 24 hpi. To determine if TRIM32 plays an antibacterial and immunological function in *Lm* infection, we infected wild-type (WT) and *Trim32*^−/−^ mice (Additional file 1: Fig S1) with *Lm* and followed their survival rate over time (Fig. 1B). All WT mice died at 7 days post infection (dpi), whereas only half of *Trim32*^−/−^ mice perished, indicating that *trim32* deletion had a protective impact during *Lm* infection. To further confirm the involvement of TRIM32 in antimicrobial immunity against *Lm* infection, we infected WT and *Trim32*^−/−^ mice via i.p. infection and measured bacterial load at 1 and 3 dpi in the spleen and liver, which were the major organs colonized by *Lm* [18]. As shown in Fig. 1C, D, WT mice had approximately 100-fold higher *Lm* load in their livers and spleens than *Trim32*^−/−^ mice. Also, we examined the spleen white plaques and liver histopathological of *Lm*-infected mice. At 3 dpi, WT mice displayed severe white plaques in spleen (Fig. 1E) and erosion in liver (Fig. 1F), and *Trim32*^*−/−*^ mice showed relatively only minor damage. Together, the *Lm* infection and the increased expression of TRIM32 promoted bacterial growth and proliferation in mice.

### Trim32 deficiency reduced production of pro-inflammatory cytokines while increased chemokines

**Fig. 2:**
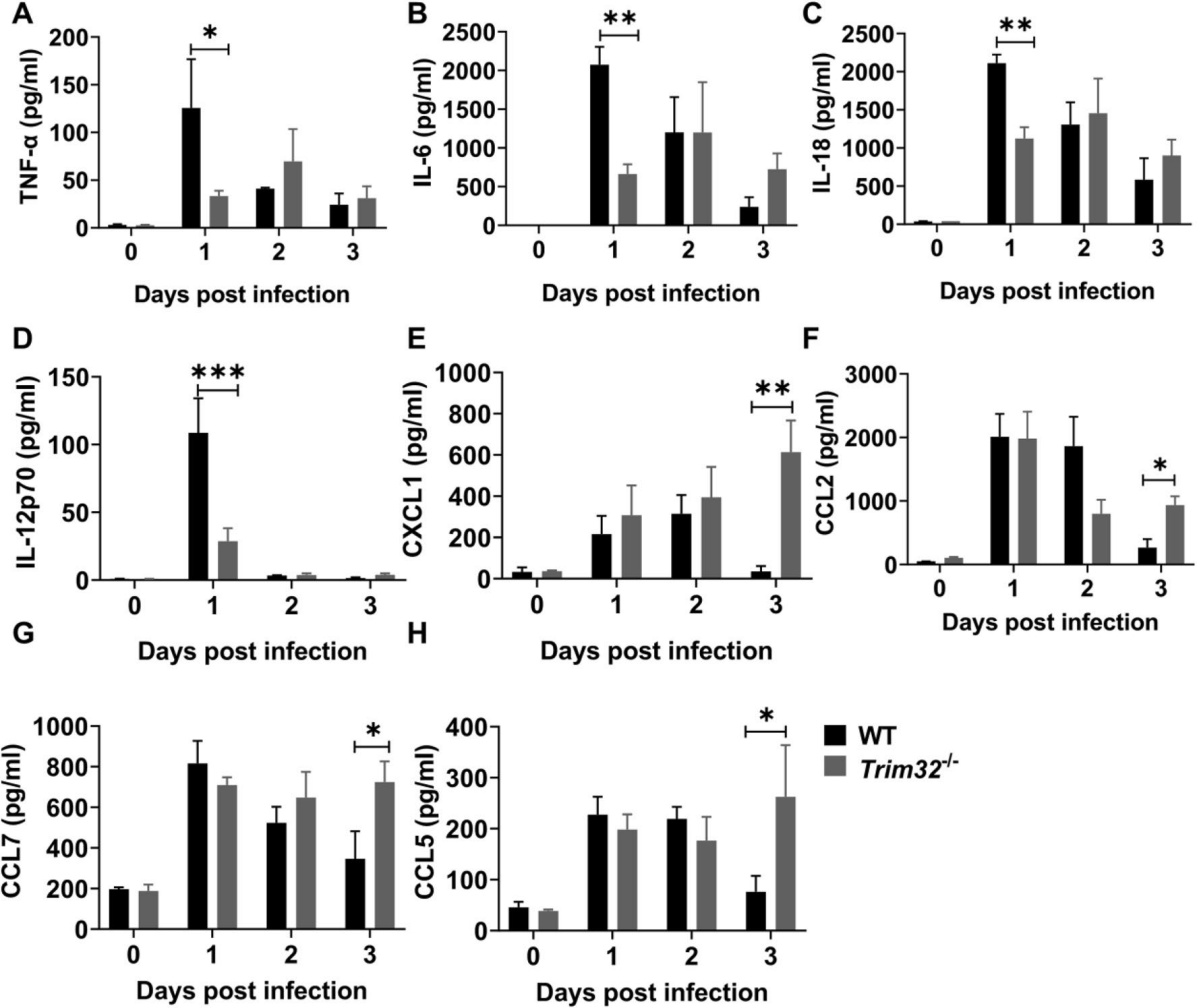
*Trim32* deficiency reduced pro-inflammatory cytokine production while increased chemokine production. *Trim32*^−/−^ and WT mice were i.p. injected with 1.0 × 10^6^ CFUs of *Lm*. Levels of pro-inflammatory cytokines (**A**–**D**) and chemokines (**E**–**H**) in sera of mice at indicated time points were measured using Luminex. Shown were mean ± SEM of 3 mice per experimental group. Data were a representative of three independent experiments. Statistical significance was calculated by means of two-way ANOVA followed by Dunnett’s multiple comparisons test; * *P* < 0.05, ** *P* < 0.01

We evaluated serum cytokine levels in WT and *Trim32*^−/−^ mice at 0, 1, 2, and 3 dpi, as cytokines have previously shown to be involved in the early pathogenesis of *Lm* infection and disease progression. Compared to WT mice, the proinflammatory cytokines IL-12, IL-18, IL-6, and TNFα were significantly reduced in *Trim32*^−/−^ mice at 1 dpi (Fig. 2A–D), whereas the chemokines CXCL1 (neutrophil trafficking, Fig. 2E), CCL2 and CCL7 (monocytes trafficking, Fig. 2F, G), and CCL5 (macrophages trafficking, Fig. 2H) were significantly increased in *Trim32*^−/−^ mice at 3 dpi. As a result, TRIM32 promoted the secretion of pro-inflammatory cytokines while inhibiting the secretion of chemokines in mice sera during the early stages of *Lm* infection.

### Trim32 deficiency reduced production of IFN-β and IFN-γ

**Fig. 3:**
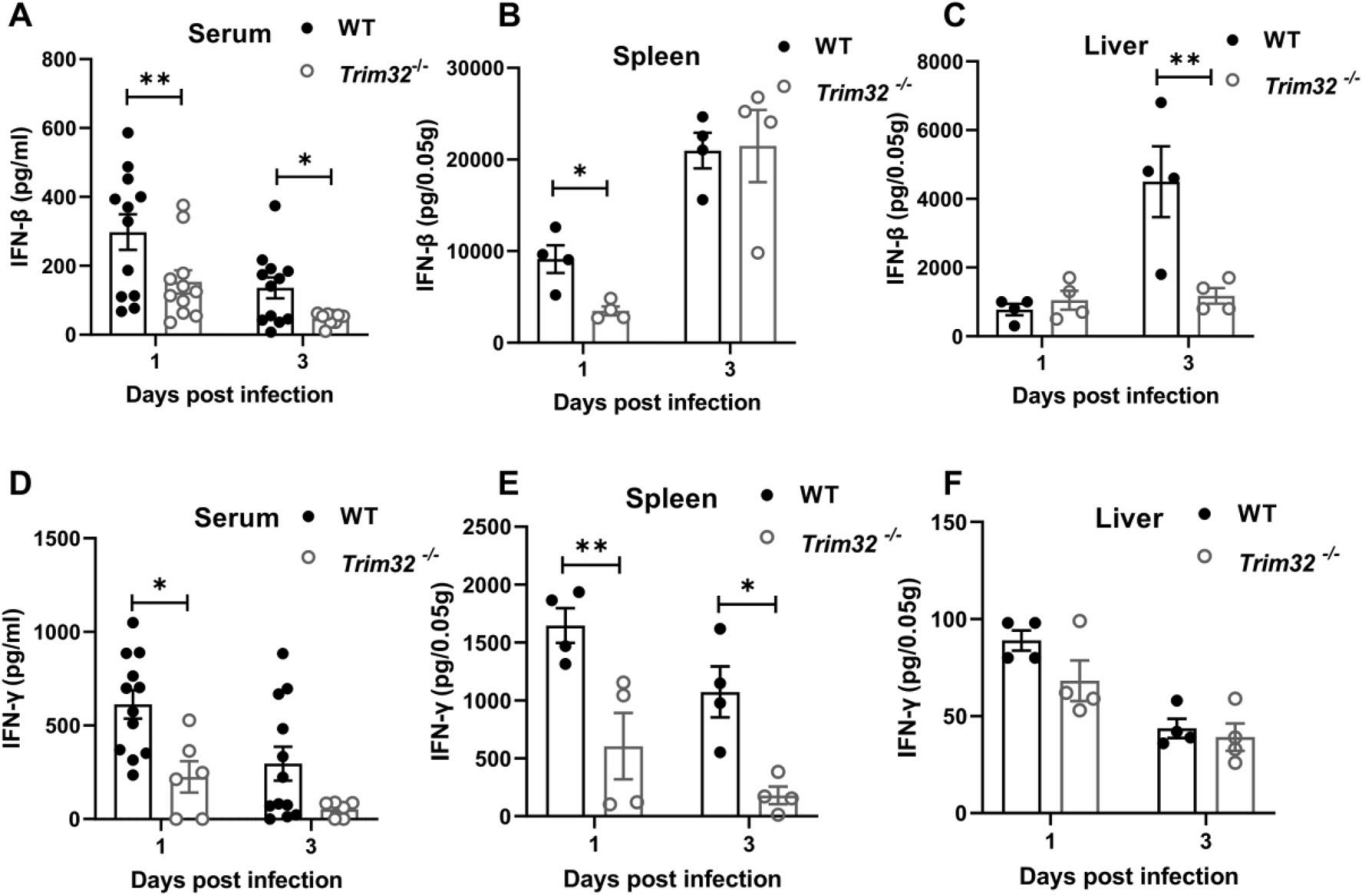
*Trim32* deficiency reduced IFN-β and IFN-γ production. *Trim32*^−/−^ and WT mice were i.p. injected with 1 × 10^6^ CFUs of *Lm*. Levels of IFN-β **A**-**C** and IFN-γ **D**-**F** in serum and in supernatants of homogenized spleens and livers at indicated time points were determined using ELISA. **A**, **D** Showed were the combined results of two independent experiments with a total of 10 mice per experimental group and time point **B**, **C**, **E**, **F**. Data were a representative of three independent experiments. Each symbol represented one mouse. Statistical significance was calculated by means of two-way ANOVA followed by Dunnett’s multiple comparisons test; * *P* < 0.05, ** *P* < 0.01

We measured IFN-β and IFN-γ in serum, spleens and livers of WT and *Trim32*^−/−^ mice at 1 and 3 dpi, respectively. IFN-β was significantly lower in *Trim32*^−/−^ mice serum at 1 and 3 dpi (Fig. 3A), spleens at 1 dpi (Fig. 3B), and livers at 3 dpi (Fig. 3C) compared to WT mice. Furthermore, IFN-γ was also significantly reduced in *Trim32*^−/−^ mice serum at 1 dpi (Fig. 3D), spleens at 1 and 3 dpi (Fig. 3E), and livers (Fig. 3F). Overall, our findings showed that TRIM32 increased the production of IFN-β and IFN-γ in mice following *Lm* infection.

### Trim32 deficiency enhanced recruitment of macrophages and neutrophils but decreased that of DCs

**Fig. 4:**
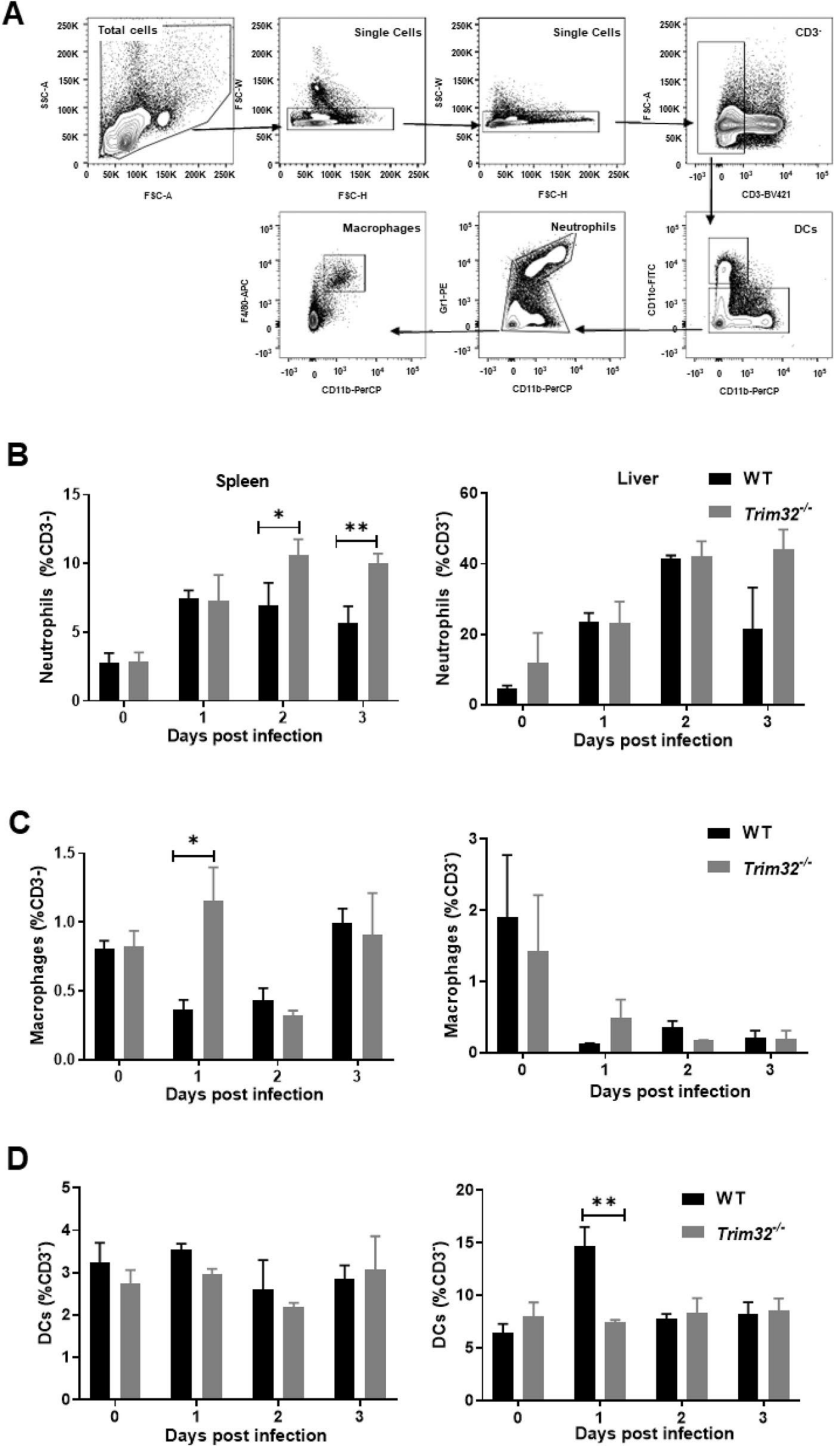
*Trim32* deficiency enhanced recruitment of macrophages and neutrophils but decreased that of DCs. **A** Schematic illustration of the gating strategy. (B-D) Relative proportions of neutrophils, macrophages, and DCs in spleen and liver. *Trim32*^−/−^ and WT mice were i.p. injected with 1 × 10^6^ CFUs of *Lm*. Mice were sacrificed at 0, 1, 2, and 3 dpi to analyze composition of innate immune cells. Shown were mean ± SEM of 3 mice per experimental group. Data were a representative of three independent experiments. Statistical significance was calculated by means of **B**–**D** two-way ANOVA followed by Dunnett’s multiple comparisons test; * *P* < 0.05, ** *P* < 0.01

Given that bacterial elimination at early stages of *Lm* infection mainly relied on innate immune cells such as macrophages, neutrophils and DCs [19], we measured the abundance of these three cells in the spleen and liver of *Trim32*^−/−^ and WT mice at 0, 1, 2, and 3 dpi. The gating strategy analyzed by flow cytometry was shown in Fig. 4A. Uninfected *Trim32*^−/−^ and WT mice had similar populations of innate immune cells in the spleen and liver (Additional file 1: Fig S2). *Trim32*^−/−^ mice had higher proportions of Gr1^+^ CD11b^+^ neutrophils in the spleen than WT mice at 2 and 3 dpi (Fig. 4B), and there was an increased trend in the liver at 3 dpi, but no statistical difference (Fig. 4B). *Trim32*^−/−^ mice had more F4/80^+^ CD11b^+^ macrophages in their spleens than WT mice at 1 dpi (Fig. 4C). Similarly, at 1 dpi, there was an increased trend in liver but no statistical difference (Fig. 4C). *Trim32*^−/−^ mice had a lower proportion of CD11b^+^ CD11c^+^ DCs in their livers at 1 dpi than WT mice (Fig. 4D). Overall, TRIM32 inhibited the recruitment of macrophages and neutrophils but promoted that of DCs at early stages of *Lm* infection.

### Trim32 deficiency increased macrophage-associated iNOS production

**Fig. 5:**
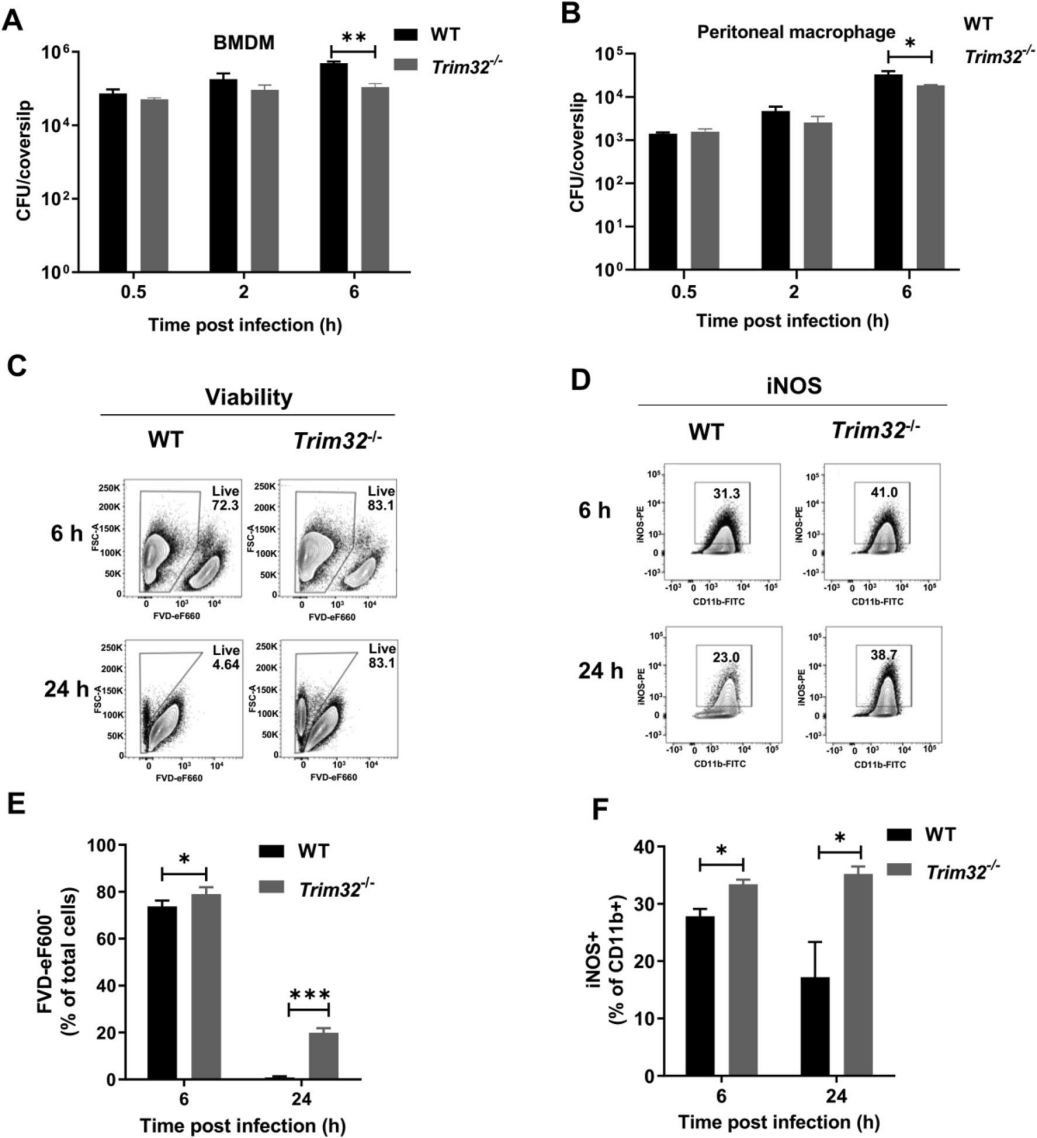
*Trim32* deficiency increased macrophage bactericidal activity. **A**, **B** Growth of *Lm* in macrophages. Macrophages were seeded in 12 mm coverslips in 24-well culturing plates and infected with *Lm* at a MOI of 0.25 for 0.5 h. *Lm* media was then discarded and replaced with DMEM containing 50 μg/mL gentamicin, then the mixture was incubated for 1 h. Bacterial counts were determined for infected BMDMs (**A**) and PMs (**B**) at 0.5, 2, and 6 hpi. Shown were mean ± SEM of 3 mice per experimental group. Data were a representative of three independent experiments. **C**, **D** Representative flow cytometry plots. BMDMs were infected by *Lm* at a MOI of 0.1 for indicated times, and then viability (**C**) and iNOS expression (**D**) in BMDMs were analyzed by flow cytometry. **E**–**F** Statistical analysis of flow cytometry data from multiple replicates. BMDMs were infected by *Lm* at a MOI of 0.1 for indicated times, and then viability of BMDMs (**E**), iNOS expression in CD11b^+^ BMDMs (**F**). Data were a representative of three independent experiments. Statistical significance was calculated by means of **A**, **B** unpaired two-tailed Student’s t-test and **E**–**F** two-way ANOVA followed by Dunnett’s multiple comparisons test. * *P* < 0.05, *** *P* < 0.001

To begin, we infected two primary mouse macrophages, BMDMs and PMs, with *Lm* at a MOI of 0.25 and then measured the intracellular bacterial growth at 0.5, 2 and 6 hpi (Fig. 5A, B). *Trim32*^*−/−*^ macrophages had a lower bacterial load at 6 hpi than WT. Furthermore, we examined cell viability and the iNOS production in BMDMs following *Lm* infection (Fig. 5C, D). FVD-eF600 (a marker of cell viability) expression was decreased in *Trim32*^−/−^ BMDMs at 6 and 24 hpi, while iNOS was increased at 6 and 24 hpi. In all, TRIM32 inhibited bactericidal activity by decreasing iNOS production in macrophages.

## Discussion

The role of TRIM32 in the pathogenesis of *Lm*-induced sepsis in mice was investigated in this study. Our findings showed that TRIM32 was induced to be expressed in mice after *Lm* infection, whereas deletion of the *Trim32* gene in mice resulted in increased animal survival and decreased bacterial load in the major infected organs, which was later linked to the down-regulation of serum proinflammatory cytokines (TNF-α, IL-6, IL-18, and IL-12p70) and interferons (IFN-β and IFN-γ). *Trim32* deficiency also increased neutrophil and macrophage recruitment to the spleen following *Lm* infection. Also, we discovered that *Trim32* deficiency increased BMDMs bactericidal activity and macrophage iNOS production. Collectively, TRIM32 aggravated *Lm-*induced sepsis in mice at early stages of infection, which was associated with increased cytokine production, decreased leucocyte recruitment and iNOS production.

Excessive serum inflammatory cytokines such as IL-6, TNF-α, and IL-18 damage host homeostasis during the early stages of innate immune defense, eventually leading to multi-organ failure and death [20, 21]. Enhanced IFN-β production not only contributes to the above-mentioned excessive cytokine production, but also facilitates *Lm* proliferation in mice [7], and mice lacking IFN-β receptor or with IFN-β production deficiency are more resistant to *Lm*-induced infection in mice [7, 22]. Similar to IFN-β, excessive IFN-γ production has a negative impact on mouse survival after *Lm* infection: *Lm*-induced infection causes excessive IFN-γ production, which inhibits granulocyte recruitment to infected sites in mice, but when mice are rejected with an IFN-γ-neutralizing monoclonal antibody, mouse survival rates improve [23]. The findings support the link discovered in this study between TRIM32 and *Lm*-induced sepsis in mice: inducible expression of TRIM32 in response to *Lm* infection results in excessive production of inflammatory cytokines and interferons, which may be the primary cause of acceleration of multi-organ lesion and increased bacterial loads. Furthermore, the increased chemokines expression in *Trim32*^*−/−*^ mice on day 3 may facilitate recruitment of immune cells, such as neutrophils and macrophages, thus contribute to the increased *Lm* bactericidal ability and survival rate. Moreover, increased iNOS expression which required to clear intracellular *Lm* infection [24–26] in macrophages maybe another major reason to increase *Trim32*^*−/−*^ bactericidal ability. In brief, low cytokines and high chemokines and iNOS expression may help *Trim32*^*−/−*^ mice resist the attack of *Lm*.

Inflammasomes activation is a well-known function of the innate immune response to *Lm* [27], leading to activates both the NLRP3 and the AIM2 inflammasome, thus leading to the secretion of IL-1β and IL-18 [28]. The roles of TRIM family members in regulating inflammasome activation are crucial in manipulating this process. Several TRIM proteins have been found to regulate NLRP3 inflammasome activation. TRIM33 positive regulates NLRP3 inflammasome activation upon stimulation with cytosolic dsRNA and bacterial RNA through the ubiquitylates dsRNA sensor DHX33 via K63 specific linkage, inducing the cleavage of caspase-1 and maturation of IL-1β and IL-18 [29, 30]. TRIM21 triggers the activation of NLRP3 by targeting STING for K63-linked ubiquitination at K20/150/224/236 through its E3 ubiquitin ligase activity, which promoted the interaction of STING with TBK1 upon RNA and DNA virus infection [31]. Similarly, TRIM32 is also an E3 ubiquitin ligase [32], but the role of TRIM32 on *Lm*-triggered inflammasome activation remains elucidated. In this study, we found decreased IL-18 level in *Trim32*^−/−^ mice serum upon *Lm* infection, suggesting a potential correlation between TRIM32 and inflammasomes. The further study will unpack the function of TRIM32.In a mouse model of *Salmonella typhimurium* (*St*) infection [33], deletion of the *trim32* gene reduced animal survival and decreased bacteria burden in the spleen and liver; these findings are contradictory and consistent with those for *Lm*-induced infection, respectively. The above contradiction may be due to the fact that *St*, as a Gram-negative bacterium, and *Lm*, as a Gram-positive bacterium, respectively, stimulate host immune response via toll-like receptor 4 (TLR4) [34, 35] and TLR2 [36, 37]. TRIM32 has been shown to ubiquitinate STING, thereby activating downstream signaling pathways such as NF-B and IRF3 [32, 38]. TRIM32 inhibits inflammatory gene transcription after *St* infection via TLR4/Myd88/TRIF-activated downstream LC3-associated selective autophagy [33]. *Lm* infection activates the TLR2-NF-κB signaling pathway to promote the production of inflammatory cytokines [39]. It is thus hypothesized that TRIM32 contributes to increased production of inflammatory cytokines in response to *Lm* infection, most likely via the TLR2-NF-κB pathway.

Overall, our findings show that TRIM32 modulates innate immune response and iNOS production in macrophages, assisting the host in fighting *Lm*-induced sepsis at the early stages of infection. TRIM32 is an intriguing host therapeutic target for the treatment of *Lm*-induced sepsis.

## Supplementary Information

**Additional file 1: Figure S1.** Validation of Trim32 knockout. (A) Diagram of trim32 knockout. (B) Gene sequencing validation of trim32 knockout. (C) Western blot validation of trim32 knockout. Data were a representative of three independent experiments. **Figure S2.** Trim32 deficiency did not affect the components of innate immune cells. (A) Schematic illustration of the gating strategy. (B) Relative proportions of innate immune cells in the spleen and liver. Shown were mean ± SD of 3 mice per experimental group. Data were a representative of three independent experiments

## Data Availability

The original data for this study are available from the corresponding author.
