# Supplementary figures and images for: TRIM32 reduced the recruitment of innate immune cells and the killing capacity of Listeria monocytogenes by inhibiting secretion of chemokines
Source: Gut Pathog. 2023 Jul 6;15:32. doi: 10.1186/s13099-023-00558-9 (PMC10324126; doi:10.1186/s13099-023-00558-9)

**Figure S1**

**
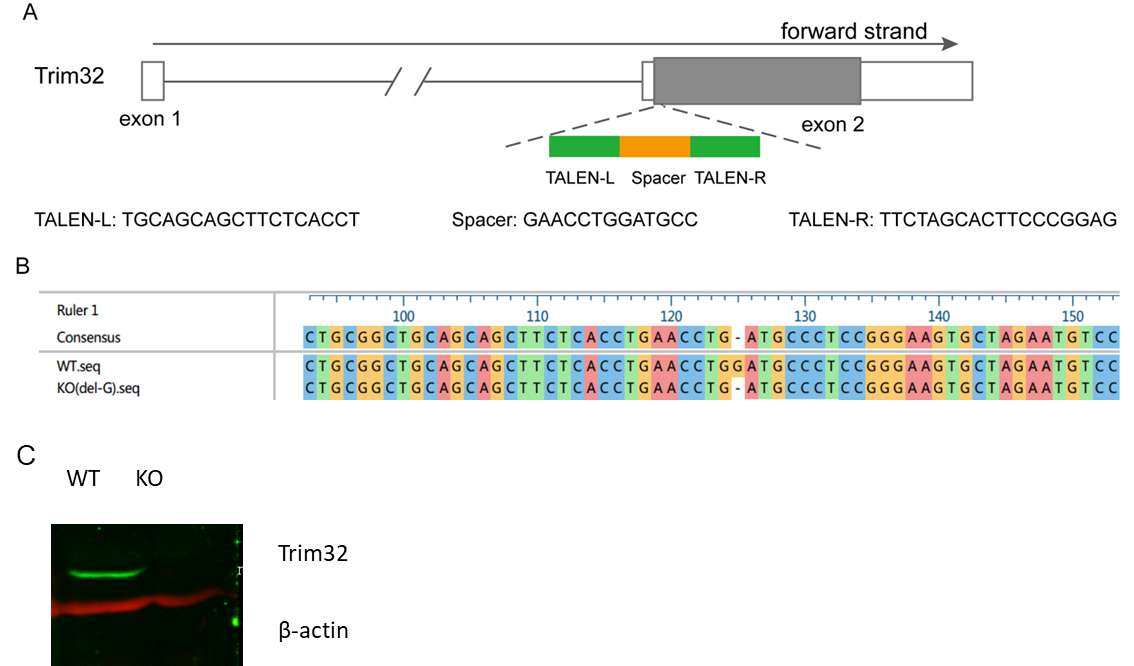
**

**Figure S2**


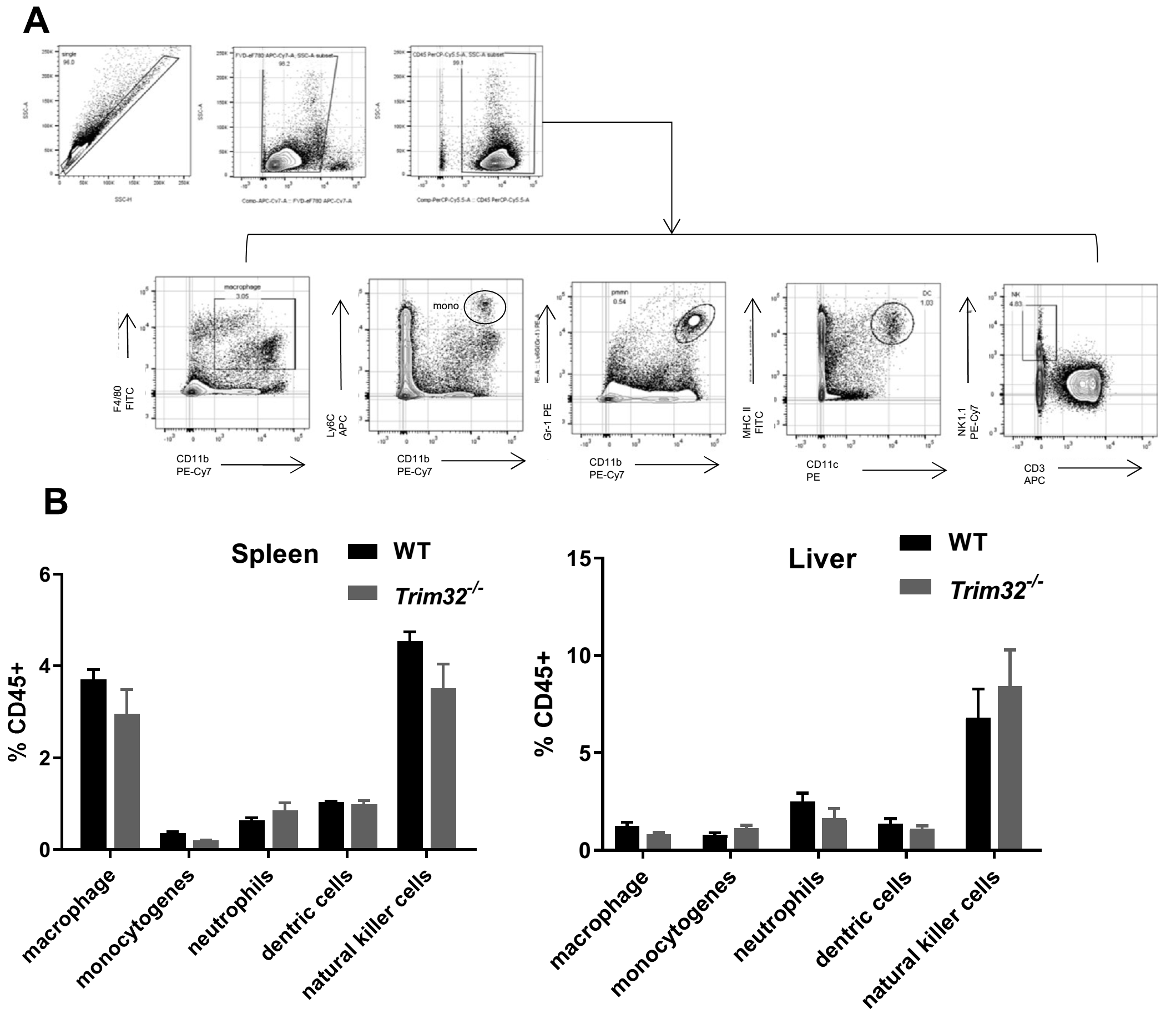

Supplement: Supplementary file 1 — Additional file 1: Figure S1. Validation of Trim32 knockout. (A) Diagram of trim32 knockout. (B) Gene sequencing validation of trim32 knockout. (C) Western blot validation of trim32 knockout. Data were a representative of three independent experiments. Figure S2. Trim32 deficiency did not affect the components of innate immune cells. (A) Schematic illustration of the gating strategy. (B) Relative proportions of innate immune cells in the spleen and liver. Shown were mean ± SD of 3 mice per experimental group. Data were a representative of three independent experiments [file 13099_2023_558_MOESM1_ESM.docx]
